# Efficiency of Wood-Dust of *Dalbergia sisoo* as Low-Cost Adsorbent for Rhodamine-B Dye Removal

**DOI:** 10.3390/nano11092217

**Published:** 2021-08-28

**Authors:** Dibyashree Shrestha

**Affiliations:** Department of Chemistry, Patan Multiple Campus, Tribhuvan University, Patan Dhoka, Lalitpur 44613, Nepal; dibyashreeshrestha@gmail.com

**Keywords:** activated carbon, *Dalbergia sisoo*, rhodamine B, contact time, contact temperature, pH

## Abstract

Wood-dust of *Dalbergia sisoo* (Sisau) derived activated carbon (AC) was successfully tested as an adsorbent material for the removal of rhodamine B dye from an aqueous solution. The AC was prepared in a laboratory by the carbonization of wood powder of *Dalbergia sisoo* at 400 °C in an inert atmosphere of N_2_, which was chemically activated with H_3_PO_4_. Several instrumental techniques have been employed to characterize the as-prepared AC (Db-s). Thermogravimetric analysis (TGA)/differential scanning colorimetry (DSC) confirmed that 400 °C was an appropriate temperature for the carbonization of raw wood powder. The FTIR spectra clearly confirmed the presence of oxygenated functional groups such as hydroxyl (–OH), aldehyde/ketone (–CHO/C=O) and ether (C–O–C) at its surface. The XRD pattern showed the amorphous structure of carbon having the 002 and 100 planes, whereas the Raman spectra clearly displayed G and D bands that further confirmed the amorphous nature of carbon. The SEM images displayed the high porosity, and the BET analysis revealed a high surface area of 1376 m^2^ g^−1^, a pore volume of 1.2 cm^3^ g^−1^, and a pore size of 4.06 nm with the coexistence of micropores and mesopores. The adsorption of dyes was performed by varying the dye concentration, pH, time, and the sample dose. The maximum percent of RhB dye removal by AC (Db-s) was 98.4% at an aqueous solution of 20 ppm, pH 8.5, an adsorbent dose of 0.03 g, and a time of 5 min. This study proved to be successful in addressing the local problem of wastewater pollution of garment and textile industrial effluents using locally available agro-waste of *Dalbergia sisoo*.

## 1. Introduction

In present days, effluents discharged from various industries, such as textiles, paints, printing, cosmetics, pulp mills, paper, rubber, pharmaceuticals, plastics, foods, and leather, have become a universal problem. These industries extensively use a large volume of dyes and generate plentiful wastewater which pollutes the environment. They have been rated as the most infamous and unadorned pollutants amongst all other sectors owing to the fact that textile industries are the major dye consumers in each of their products [1]. Within dyes, textile dyes are one of the chief components among them [2] and, unfortunately, have a harmful effect on human and animal health [3]. These pollutant dye compounds change the color of water, and it makes the degrading quality of water for both flora and fauna [4]. It is reported that about 100,000 commercial dyes are in industry and larger than 7 × 10^5^ tons of dyestuff are produced annually. The issue of enormous amounts of synthetic dyes to the water streams has stood a serious risk to the environment [5]. A very small amounts of dyes in water (less than 1 ppm for some dyes) is highly visible and undesirable [6].

Most dyes molecules are complex and stable and can be resistant to degradation upon contact with water, detergents, or any other thing [7]. In order to remove dyes from wastewater, a wide range of techniques, such as adsorption, coagulation and flocculation, biological treatment, ion exchange, membrane filtration chemical oxidation, reverse osmosis, and electrochemical processes [8], have been employed. Among all, adsorption technology, being very easy, economical, effective and flexible, has become the most preferred methods for the elimination of toxic and hazardous dyes from wastewater.

To greatly decease the cost of activated carbon (AC), various ACs prepared from non-conventional sources, such as coir pith [9], sawdust and rice husk [10], and pinewood [11], applied in the removal of some compounds have also been investigated, and better results have been attained.

The yield and absorptive property of AC is increased by using various suitable activating agents, such as H_3_PO_4_, KOH, H_2_SO_4_, and Na_2_CO_3_. Among them, the AC prepared from H_3_PO_4_ has been reported to be the best in terms of active surface areas and porosity and functional groups. In this study, phosphoric acid (H_3_PO_4_) has been used as an activating agent to open pores and enlarge the surface area of AC, so that the adsorption power will increase [12]. H_3_PO_4_ removes impurities contained in the carbon [13], as a result of the carbonization process of biomass such as ketones, alcohols, acids, and aldehydes. Moreover, H_3_PO_4_ also helps to form a bridge that connects the fragments of biopolymers (layer of carbon structure) by forming phosphate bonds that make pores more open and enlarge the surface area. In the present study, basic/cationic water-soluble dyes rhodamine B (RhB), which is carcinogenic in nature, was selected for the adsorption experiment due to its presence in wastewaters from by-products of several industries such as textile, leather, jute, and food industries.

RhB is an amphoteric dye, listed in the class of the xanthene dye causing harmful effects such as acute oral toxicity. When consumed, it causes damage to the eyes or skin irritation and is hazardous to the aquatic organism with long-term effects. Therefore, the treatment of effluent containing RhB dye becomes paramount before discharging them into water streams so as to protect the aquatic organism and make the environment safer for the public [14]. 

In addition, AC is amongst the most used adsorbents utilized due to its high surface area, pore volumes, fast adsorption rate, and high adsorption capacity. Herein, AC from *Dalbergia sisoo* has been prepared by chemical activation with H_3_PO_4_ [15]. The porous properties, chemical functionality, and surface morphology of the prepared ACs are performed by N_2_ adsorption, FTIR, and SEM [16].

It is found from previous research that ACs modified by different surfactants have different effects on various water qualities of dye wastewater. Therefore, it is necessary to determine the coexistence of other ions that would affect the adsorption behavior. Meanwhile, more information is still required in order to better understand the adsorption behavior of RhB cationic dyes on the modified AC. The physical and chemical characteristics of RhB is given in Table 1. The aim of this study was to examine the potential of using the as-prepared AC from wood powder of *Dalbergia sisoo* tree as a low-cost adsorbent to remove RhB dye from aqueous solutions using UV-Vis spectrophotometry. The effect of the precursor dose and time of adsorption was the central focus of this study.

## 2. Materials and Methods

### 2.1. Reagents

All the chemicals used in this study were of analytical grade and were used without further refinement. Phosphoric acid (85%) was obtained from Fischer Scientific, Pune, Maharashtra, India. Similarly, RhB (98%) was procured from Alfa Aesar A13572 (Haverhill, MA, USA), and aqueous NH_3_ (30%) were from Baker (Anford, ME, USA). Double-distilled water was used throughout the experimental work. The wood dust of *Dalbergia sisoo* (Sisau) was collected from indigenous carpentry/sawmill, Kathmandu, Nepal. A commercial carbon made from coconut shells having a surface area of 876.02 m^2^ g^−1^, a pore size of 4.23 nm, and a pore volume of 0.19 cm^3^ g^−1^ was also used for comparing the results.

### 2.2. Methods

#### 2.2.1. Preparation of Wood Powder

Wood dust was sun-dried for a few days. Then, a few initial stages were done, such as crushing, grinding, and sieving. The particle size was controlled by sieving through a 150 µm sized sieve. Thus, the obtained fine wood powder was used as a precursor for the preparation of AC.

#### 2.2.2. Preparation of AC by the Carbonization Method

Wood dust (precursor) powder was dried in an oven to remove all volatile matters. The dried precursor was chemically activated with H_3_PO_4_ using the ratio of precursor to activating agent of 1:1 (*w*/*w*), left for overnight at room temperature for proper soaking and then evaporated to dry at 110 °C in an oven. The weighed amount of samples were inserted into a horizontal electric tubular furnace tube and carbonized at 400 °C for 3 h in an inert atmosphere of N_2_. A continuous flow of pure N_2_ was used to create an inert atmosphere. The carbonized samples were then cooled to room temperature maintaining the inert atmosphere of nitrogen and were washed with distilled water for several times until the pH of the washed water became neutral. It was dried, ground and stored in an air-tight container. The resulting material was dried in an oven at 110 °C and named as AC (Db-s). Thus, the prepared AC (Db-s) was stored in an air-tight container until further use in adsorption experiments.

**Scheme 1:**
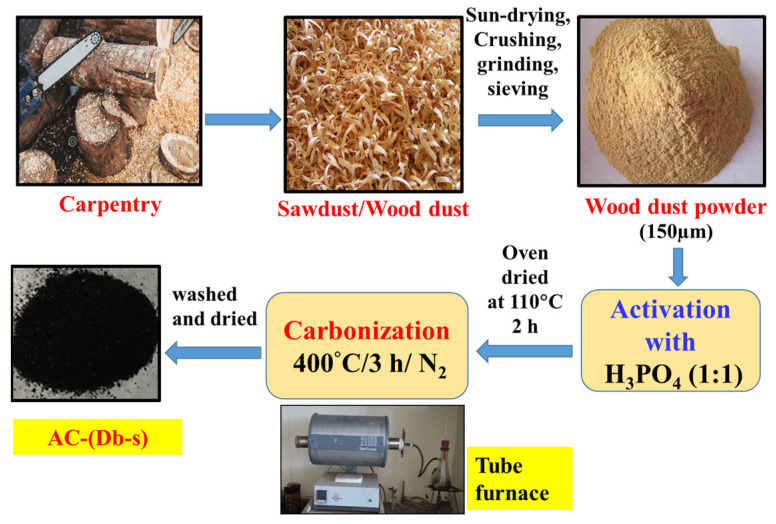
Steps involved in the preparation of activated carbon from *Dalbergia sisoo.*

A schematic diagram for the preparation of AC (Db-s) followed by carbonization is shown below in Scheme 1.

The descriptions of the as-prepared AC (Db-s) and conditions are given in Table 2.

#### 2.2.3. Characterization Technique of the As-Prepared AC (Db-s)

The moisture content, ash content, volatile matter, and fixed carbon of the precursor were analyzed by proximate analysis. The pyrolytic behaviors of the raw wood powder of *Dalbergia sisoo* were investigated by thermogravimetric analysis (TGA)/differential scanning colorimetry (DSC; SDT Q600 V20.9 Build 20). The morphology of AC (Db-s) was determined by a scanning electron microscope (Nanoeye, Korea). The phase state was evaluated by using XRD (Rigaku RINT 2000 diffractometer). Similarly, surface carbon-oxygen functional groups present in the samples were identified performing FTIR measurements using an FTIR instrument (Bruker, Vertex 70, Germany). The % transmission of the samples was recorded between 4000 and 400 cm^−1^. The presence of the amorphous nature of AC (Db-s) was confirmed by Raman signals obtained by the labRAM HR800 (JOBIN YVON). The surface area, pore size, and pore volume of the AC (Db-s) were measured by BET (Micromeritics ASAP 2020 system).

#### 2.2.4. Methodology of Dye Adsorption

A 20 ppm stock solution of RhB was been prepared by dissolving 0.02 g of the dye per liter of double-distilled water. At first, a 100 mL RhB dye solution was taken in 250 mL Erlenmeyer flasks, and 0.02 g of the as-prepared AC (Db-s) was added and stirred at 400 rpm at neutral pH for 5 min on a magnetic stirrer at room temperature. During the stirring period, about 3 mL of the mixture solution were taken in a microcentrifuge tube in every single minute time interval for 5 times. All the 5 microcentrifuge tubes were then centrifuged for 5 min to separate the insoluble particles. The agitation speed was kept constant at 400 rpm.

## 3. Results and Discussion

### 3.1. Proximate Analysis

The results obtained from the proximate analysis of precursor (wood powder of *Dalbergia sisoo*) are given in Table 3. The low ash content in the samples indicated that the precursor contained low inorganic matter, and the low moisture content exhibited the raw material is good for the preparation of AC and the efficiency of reactivation.

### 3.2. TGA/DSC

**Figure 1:**
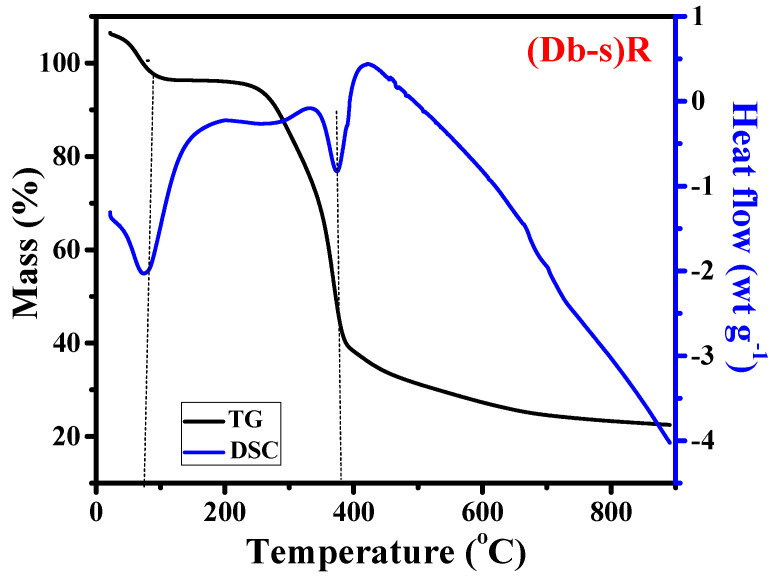
Thermogravimetric (TG) and differential scanning colorimetry (DSC) curves of raw wood powder of *Dalbergia sisoo* (Db-s) R.

The pyrolytic behavior of the wood powder of *Dalbergia sisoo* (Sisau) was measured by TGA/DSC. Figure 1 Illustrates the thermogravimetric (TG) and DSC curves of the raw wood powder of *Dalbergia sisoo* [(Db-s) R].

A minor mass loss was detected at around 100 °C in TG curves which might be due to dehydration, which was again confirmed by a sharp peak of the DSC curve at 100 °C. Similarly, at around 200–300 °C, there was a contracted peak, which was not clear in the TG plot. However, from the DSC curve, minor mass loss was clearly detected between 200 and 300 °C, which might be due to the breakdown of hemicellulose which was noticeably completed at 300–310 °C.

A significant mass loss was detected between 300 and 400 °C from the TG curve, which was confirmed by peak at around 390 °C of the DSC curve. The breakdown of cellulose started at around 300 °C and by 400 °C, and all cellulose changed to organic volatile matters present in raw wood powder. It exhibited that more than 60% of weight loss occurred around 400 °C [17]. The samples were more stable beyond 400 °C. Accordingly, 400 °C is an appropriate temperature for the carbonization process [18].

### 3.3. XRD Measurement

**Figure 2:**
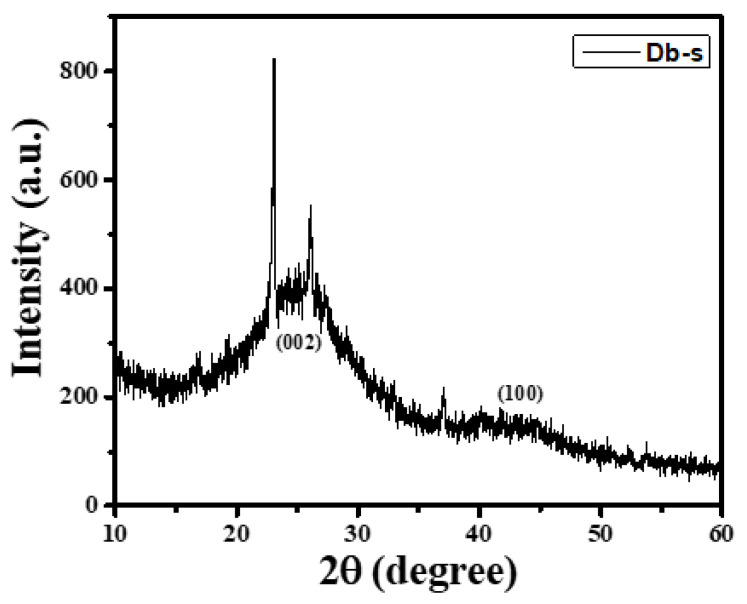
XRD pattern of AC (Db-s).

XRD analysis was carried out in order to determine the degree of crystallinity or amorphous nature of the as-prepared AC (Db-s). The XRD pattern of AC (Db-s) is presented in Figure 2.

The broad peaks at 2θ degrees of around 24 to 26 from the (002) plane and also at 2θ degrees of around 43 to 45 from the (100) plane could be clearly seen in AC (Db-s). The absence of a sharp peak and the appearance of a wide peak indicating the amorphous nature and graphitic crystallites as in commercial AC [19], in which 2 distinct peaks at 2θ degrees of 25 and 45 coming from the (002) and (100) planes of graphitic clusters, respectively, were reported. Similarly, a wide peak of graphite clusters has been reported in porous carbon by Molina-Sabio [20]. However, a few sharp peaks could be seen in Ac (Db-s), which might be due to some impurities or moisture present in the samples.

### 3.4. Raman Scattering Analysis

**Figure 3:**
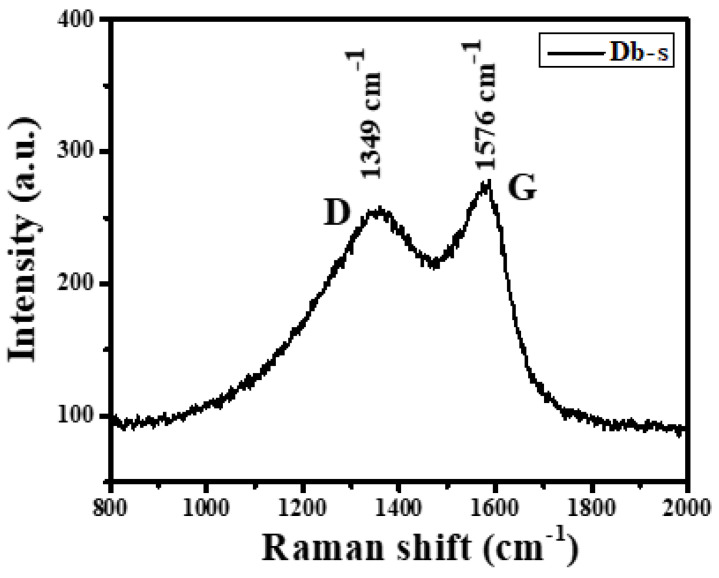
Raman spectra of the as-prepared AC (Db-s).

The Raman spectra of the as-prepared AC (Db-s) are presented in Figure 3. The Raman spectra of AC (Db-s) revealed strong D and G bands at approximately 1349 and 1576 cm^−1^, respectively.

Typically, the advent of D band in Raman scattering is associated with a disordered carbon structure, and the intensity of D band is correlated with defects (amorphous). If there are a large number of defects, it will show a high intensity of D band, whereas the absence of defects correlates with the absence of D band as in case of carbon nanotubes and graphitic carbons. Hence, D band at 1349 cm^−1^ is a characteristic of disordered carbon or amorphous carbon in AC (Db-s). Therefore, this finding of Raman spectroscopy approved pleasantly with the XRD results [19].

### 3.5. FTIR Analysis

**Figure 4:**
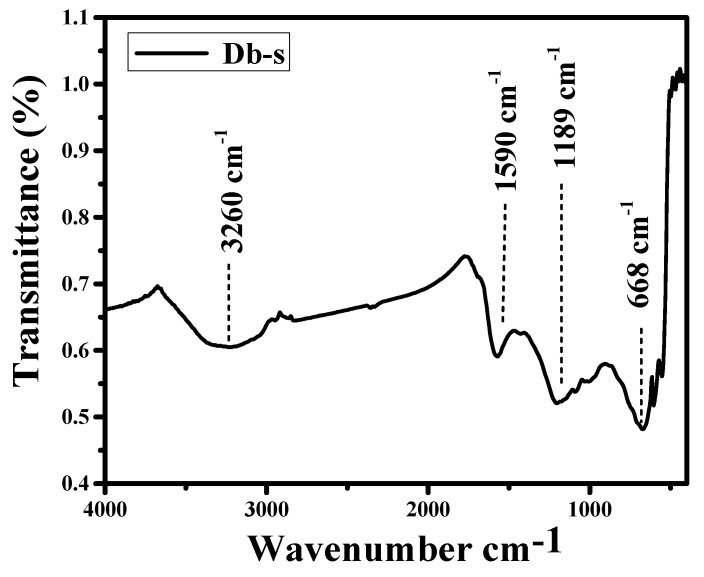
FTIR spectra of AC (Db-s).

The FTIR spectra of AC (Db-s) are shown in Figure 4. As can be seen in Figure 4, the spectra showed a strong wide adsorption band at around 3260 cm^−1^. The position of the band was characteristics of the stretching vibration of hydroxyl groups of carboxyl, phenol, and alcohol. –OH band can also be correlated with adsorbed water present in raw samples as indicated by TG/DSC analysis, where, at around 100 °C, mass loss was clearly seen, indicating the evaporation of water molecules. The band observed at the region around 1590 cm^−1^ is due to olefinic C=C vibrations in aromatic rings. The band at around 1189 cm^−1^ is due to the asymmetric stretching of C–O bond in acids, alcohols, phenols, and esters. The band at around 668 cm^−1^ is attributed to C–C stretching in a fingerprint region. These results are in good agreement with the findings of many investigators [21]. The FTIR results clearly expressed that the as-prepared AC (Db-s) was fully functionalized with oxygen containing groups such as carboxylic, phenolic, lactonic, and ether groups by the use of an activating agent (H_3_PO_4_).

### 3.6. N_2_ Adsorption/Desorption Isotherm/Brunauer–Emmett–Teller (BET) Theory 

**Figure 5:**
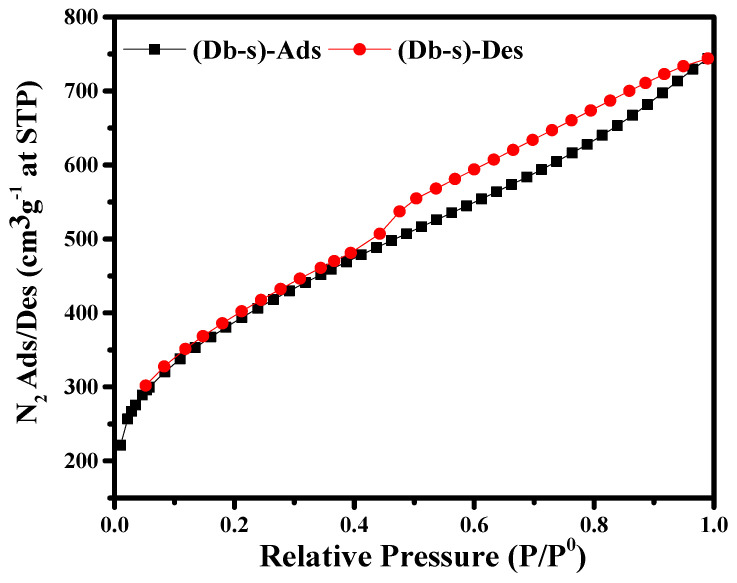
N_2_ adsorption/desorption isotherms of AC (Db-s) at 77 K.

The N_2_ adsorption/desorption isotherms of AC (Db-s) at 77 K are shown in Figure 5. As can be seen in Figure 5, Type II H_3_ isotherm could be seen. At relatively lower pressure (P/P^0^ < 0.1), the isotherms showed an insignificant uptake, indicating the presence of a few micropores in AC (Db-s).

However, at around P/P^0^ = 0.4, the amount of nitrogen uptake increased significantly, showing a hysteresis loop during the adsorption and desorption of nitrogen. The adsorption volume of AC (Db-s) increased with the relative pressure until P/P^0^ ≅ 0.5. The widely opened knees and the slight hysteresis loops at the relative pressure of 0.5 to 1.0 indicated the presence of a considerable amount of small mesopores in AC (Db-s). Therefore, from the isotherm of AC (Db-s), it can clearly be seen that the as-prepared AC sample consisted of an innumerable amount of mesoporores along with some micropores, since a hysteresis loop is the characteristics of mesopores [22].

Then, the BET specific surface area, pore size, and pore volume of AC (Db-s) were measured, and the results are given in Table 4. The large surface area, small pore size, and pore volume might be due to the presence of innumerable mesoporosity along with microporosity [20].

### 3.7. SEM Analysis

**Figure 6:**
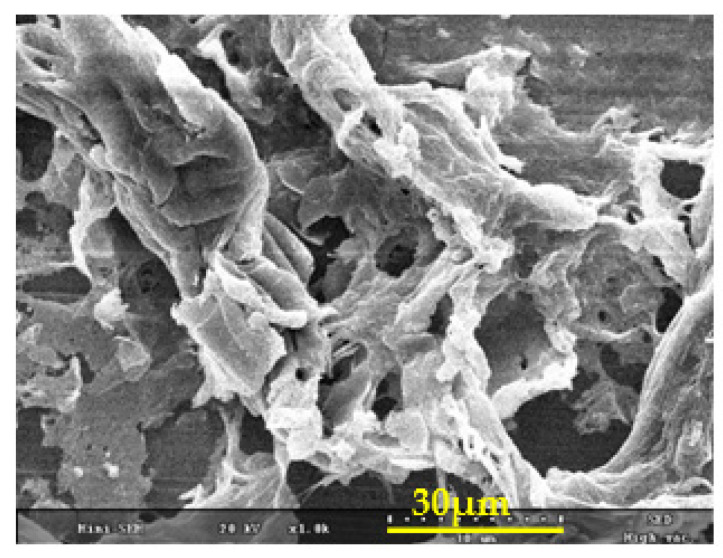
SEM image of AC (Db-s) showing a porous morphology.

Figure 6 illustrates the SEM micrographs of AC (Db-s), at s magnification of 1000, showing a well-developed porous morphology with a layered structure.

The pore development on the surfaces of AC (Db-s) might be due to the course of dehydration of an activating agent, i.e., H_3_PO_4_ [19] and then react with oxygen to generate phosphoric anhydride (P_2_O_5_). Here, P_2_O_5_ sublimated from solid to gas at 360 °C, and then, gaseous P_2_O_5_ escaped from the surface of the AC-creating pores [23]. In addition to this, during the washing process, remaining P_2_O_5_ was hydrolyzed and removed, creating an empty space or pores. Pores may be micropores or mesopores with a volume corresponding to that of the removed phosphoric acid [24]. Accordingly, the presence of incredible mesoporosity along with microporosity is expected to enhance the adsorption capacity of AC (Db-s). It is also supported by the BET results of Figure 5 and Table 4.

The adsorption capacity of AC depends on surface functional groups, surface areas, and pore sizes and morphology. As reported in the relevant literatures [24], for the adsorption of dyes from effluents, ACs with oxygen containing functional groups, high surface areas (above 1000 mg^−1^), and good morphology with extensive mesoporosity are required, because dyes are larger molecules (above 2 nm) which are fitted into the mesopores. Under the basis of these requirements, the as-prepared AC (Db-s) is considered to be well-matched for the adsorption of RhB dye.

The adsorption of dye molecules are affected by various factors such as adsorbent dose, solution pH, initial dye concentration, and contact time. The optimization of these parameters will greatly help in the research work and mainly in the development of industrial scale treatment process for the dye removal.

### 3.8. Effects of Variable Parameters

#### 3.8.1. Effect of pH and Contact Time on the Adsorption of RhB

**Figure 7:**
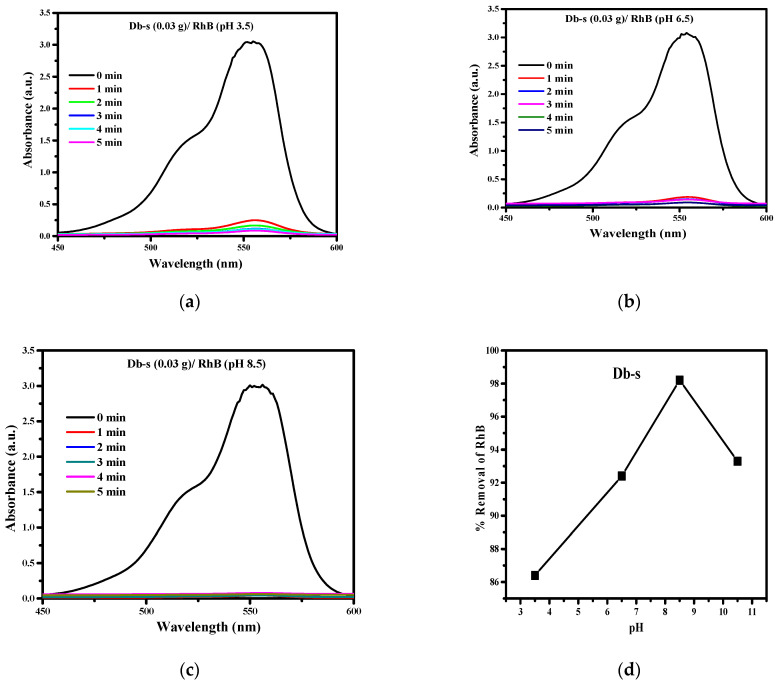
(**a**) Adsorption spectra of RhB during adsorption at pH 3.5 and at different times containing 0.03g of AC (Db-s). (**b**) Adsorption spectra of RhB during adsorption at pH 6.5 and at different times containing 0.030g of AC (Db-s). (**c**) Adsorption spectra of RhB during adsorption at pH 8.5 and at different times containing 0.03g of AC (Db-s). (**d**) Effect of pH on the removal of RhB by AC (Db-s).

The effects of pH and contact time on the adsorption of 20 ppm RhB were studied spectrophotometrically at a fixed dose (0.030 g/100 mL) of adsorbent AC (Db-s). Figure 7a–d shows the UV-Vis spectra of RhB dyes during the adsorption at pH 3.5, 6.5, and 8.5 at time intervals of 1, 2, 3, 4, and 5 min.

Here, as can be seen in Figure 7a, at pH 3.5, the adsorption of RhB dye by AC (Db-s) was not completed up to 5 min.

It is obvious in Figure 7b that, at pH 6.5, the adsorption of RhB dye by AC (Db-s) was still not completed within 5 min. However, the adsorption was higher than in the case at pH 3.5.

Figure 7c shows that at pH 8.5, almost all of RhB dyes were adsorbed by AC (Db-s) and all the adsorption spectra are in a shape of a straight line for 4 and 5 min. A similar measurement carried out at pH 11 revealed a decrease in the adsorption of RhB.

The percentage removal of RhB was estimated from Figure 7a–c at different pH values, and the effect of pH on the removal of RhB is plotted in Figure 7d. The results showed a gradual increase in % removal of RhB with pH until 8.5 and afterwards a decrease in % removal of RhB.

The less % removal (low adsorption) of RhB dye in an acidic medium (pH 3.5) can be explained by the fact that at this acidic pH the adsorbent surface acquired a positive charge which repelled the cationic dye RhB [25,26]. In addition, increased hydrogen ion (H^+^) at low pH may compete with dye ions for the adsorption on AC (Db-s). However, in an alkaline medium, i.e., at higher solutions at pH 8.5, AC (Db-s) may be negatively charged, which enhances the adsorption of positively charged dye cations through electrostatic forces of attraction.

RhB is an aromatic amino acid with amphoteric characteristics due to the presence of both the amino group (–NHR_2_) and the carboxyl group (–COOH). Thus, the charge state of RhB is dependent on the solution pH. When the pH increases higher than 8.5, the ionization of the carboxyl group takes place which is attracted to positive charges on the xanthane group and the zwitterions form of RhB is formed. The zwitterions form of RhB in water may increase the dimerization of RhB, which makes the molecule too large to enter most of the pore structure of AC. The inaccessibility to the pore structure of AC, which is smaller than the dimer’s effective size, results in a decrease in RhB removal at pH above 8.5. The variation of the RhB removal with the solution pH is similar to that reported previously [27,28,29,30].

Figure 7a–d reveals that the initial adsorption percentage of dyes was quite rapid. Almost 80% of the dyes were adsorbed within a single minute. Then, the adsorption process was more or less constant up to 5 min. This is probably due to a high surface area of 1376 m^2^ g^−1^ with extensive mesoporosity in AC (Db-s). Highly developed mesoporous ACs are favorable for the adsorption of larger molecules such as dyes [27]. This might be due to the fact that initially all the sites of the AC samples were vacant and available for dye molecules to get occupied. Later, the percentage removal/adsorption rate of dye was decreased significantly, resulting from the saturation of dye molecules on the surfaces of the AC samples.

#### 3.8.2. Optimization of the Dose of AC (Db-s)

The efficiency of adsorbent doses on the RhB dye adsorption is reported by many researchers to determine the most economical minimum dosage. In general, the dye removal percentage increases with the increase of the adsorbent dosage [28].

Here, various doses of adsorbent AC (Db-s) were mixed with a fixed amount of dye solutions, and the mixture was agitated in a mechanical shaker. The adsorption capacities for different doses were determined at 1 min interval till 5 min by keeping all other factors constant.

**Figure 8:**
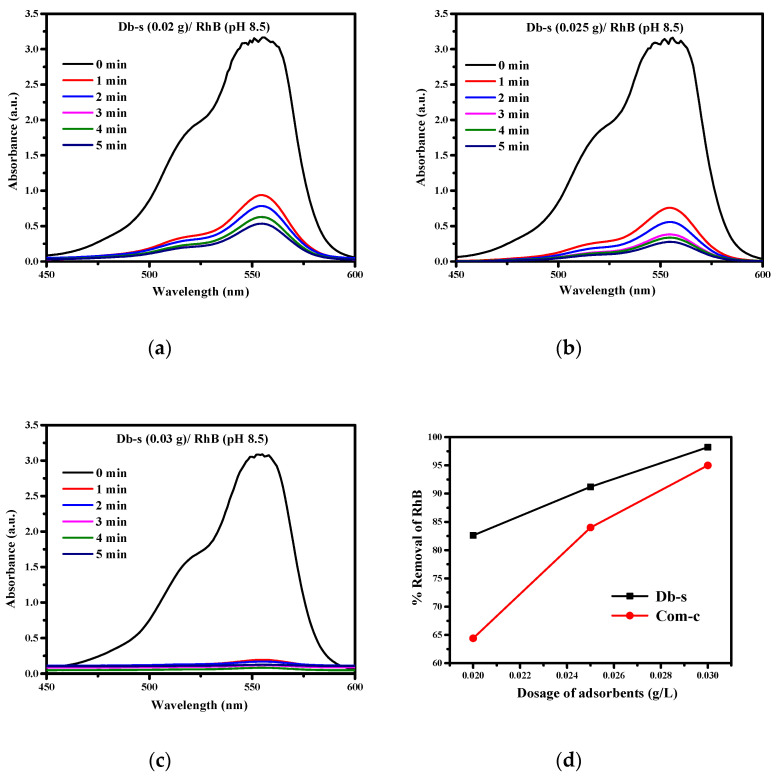
(**a**) RhB dye adsorption by AC (Db-s) at a dose of 0.02 g; (**b**) RhB dye adsorption by AC (Db-s) at a dose of 0.025 g; (**c**) RhB dye adsorption by AC (Db-s) at a dose of 0.03 g; (**d**) comparison of the % removals of RhB by AC (Db-s) and commercial carbon (at 0.030 g).

The percentage of adsorption increased with the increase in the dose of the adsorbent AC (Db-s) (Figure 8a–d), which are presented in Table 5. This is attributed to the increased surface area and availability of a more adsorption site [29].

The adsorption/removal capacity of the adsorbent (AC) is due to their porous structure and pore size distribution, and it depends on the polarity, solubility, and molecular size of the adsorbate [30]. From the adsorption spectra, it was observed that the optimum dose of adsorbent AC (Db-s) for the removal of RhB from an aqueous solution was 0.03 g/100 mL at a basic medium (pH 8.5) (Figure 8c).

As can be seen in Table 5, the percentage removal capacity of RhB increased from 82.6%, 91.2%, to 98.4% by the as-prepared AC (Db-s) with the increase of dose from 0.02 g, 0.025 g, to 0.030 g, respectively. When the AC (Db-s) dose was increased, free adsorption sites also increased and thus more dye molecules were adsorbed [31].

When the AC (Db-s) was increased to 0.035 g/100 mL, there was no significant difference in percentage adsorption/removal of dye from the dose of 0.030 g, and therefore, 0.030 g was suitable for the adsorption of dyes in this study. Table 6 summarizes the adsorption efficiency of Rhb dye at a dose of 0.030 g L^−1^.

From the above results in Table 5 and Table 6, it is clear that the data generated from experiments are valid and not highly deviated. Table 7 gives the summary of the analysis of the spectrometric method. Similarly, Table 8 presents the results of % removal of RhB dye by different doses of the AC (Db-s).

Furthermore, to study the efficiency of the as-prepared AC (Db-s), the % removals of RhB by using different doses of AC (Db-s) were compared with the same doses of commercial carbon, which are tabulated at Table 9. Since the best adsorption efficiency was exhibited by the dose of 0.030 g, the removal of RhB at this dose was again compared with that by commercial carbon, which is shown in Figure 8d. As can be seen in Figure 8d, the as-prepared AC (Db-s) revealed a better removal result than commercial carbon.

## 4. Conclusions

From the results on the removal of RhB dye, from agro waste of *Dalbergia sisoo* in this study, the following conclusions can be drawn:

1. AC was successfully prepared from the agro waste of *Dalbergia sisoo* by activating with H_3_PO_4_ followed by carbonization at 400 °C.
2. BET measurements showed the formation of a mesoporous structure with an active surface area of 1376 m^2^ g^−1^, a pore volume of 1.2 cm^3^ g^−1^, and a pore size of 4.06 nm.
3. AC (Db-s) was found to possess the best adsorption capacity at pH 8.5 and a dose of 0.030 g. Under this condition, 98.4% of 20 ppm of RhB dye was removed from an aqueous solution within 5 min.
4. The results revealed that laboratory prepared phosphoric acid AC derived from agro waste such as wood dust of *Dalbergia sisoo* can be converted into value-added materials for the removal of toxic dyes such as RhB.

## Figures and Tables

**Table 1:** Physical and chemical characteristics of rhodamine B (RhB) dye.

Dye	IUPAC Name	Chemical Formula	Chemical Structure	Mol. Wt. (gmol^−1^)	Appearance	λ_max_ (nm)
Rhodamine B	[9-(2-carboxyphenyl)-6-diethylamino3-xanthenylidene]-diethylammonium chloride	C_28_H_31_ClN_2_O_3_	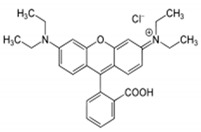	479.02	Reddish violet powder	554

**Table 2:** Description and preparation conditions of the sample.

Sample	Particle Size (µm)	Activating Agent	H3PO4:Wood Powder Ratio (*w*/*w*)	Preheating Temperature (°C)	Carbonization Temperature (°C)	Carbonization Time (h)	Atmosphere
AC (Db-s)	150	H_3_PO_4_	1:1	110	400	3	N_2_

**Table 3:** Proximate analysis of the precursor (wood powder of *Dalbergia sisoo*).

S.N.	Parameter	Observed Value (%)
1.	Moisture	8.66
2.	Total ash	1.07
3.	Volatile matter	42.27
4.	Total carbon	48.0

**Table 4:** Brunauer–Emmett–Teller (BET) specific surface area, pore size and pore volume of AC (Db-s).

Name of Sample	Specific Surface Area (m^2^ g^−1^)	Pore Size (BJH ads) (nm)	Pore Volume (BJH ads) (cm^3^ g^−1^)
AC (Db-s)	1376	4.06	1.2

**Table 5:** Adsorption efficiency of RhB dye with the surface area, pH, and doses of the samples and adsorption capacity in 5 min.

S.N.	Surface Area (m^2^ g^−1^)	Number of Adsorption	Dose (g L^−1^)	Initial Conc.(ppm)	Adsorption Capacity (%)	D@A=
1	1376	A1	0.015	20	81.5	1.225
2		A2	0.020		83.1	1.662
3		A3	0.025		91.2	2.28
4		A4	0.030		98.4	2.952
5		A5	0.035		98.4	3.444

**Table 6:** Adsorption efficiency of RhB dye at a dose of 0.030 g L^−1^.

S.N.	No. of Adsorption (N)	Dose (g L^−1^)	Initial Concentration (ppm)	Adsorption Capacity (%) (X)	X − μ	(X − μ)^2^
1	A1	0.030	20	98.4	98.4 − 98.36 = −0.04	0.0016
2	A2			98.2	98.2 − 98.36 = −0.16	0.0256
3	A3			98.5	98.5 − 98.36 = 0.14	0.0196
4	A4			98.4	98.4 − 98.36 = 0.04	0.0016
5	A5			98.3	98.3 − 98.36 = 0.06	0.0036

**Table 7:** Accuracy and precision of the spectrometric method for the determinations of RhB dye.

S.N.	Surface Area (m^2^ g^−1^)	Dose Taken (μmg L^−1^)	Dose Found (μmg L^−1^)	Mean Standard Deviation (%)	Relative Standard Deviation (%)
1	1376	15	14.78 ± 0.226	98.5	1.5
2		20	19. 84 ± 0.157	99.2	0.8
3		25	24.87 ± 0.126	99.5	0.5
4		30	29.99 ± 0.015	99.9	0.1
5		35	39.99 ± 0.012	99.9	0.1

**Table 8:** Percentage of RhB dye adsorbed by different doses of the as-prepared AC (Db-s).

S.N.	Dye	Adsorbent Dose				Result
		0.020 mg	0.025 mg	0.030 mg	0.035 mg	
1	RhB	83.1	91.2	98.4	98.4	No effect after 0.035 mg

**Table 9:** Details of the comparison of the % removal of RhB dye using different doses of AC (Db-s) and commercial carbon.

Surfactant	Surface Area (m^2^ g^−1^)	Dye	pH	Initial Conc. (ppm)	Dosage (g L^−1^)	Time (min)	% Removal
Db-s	1376	RhB	8.5	20	0.020	5	83.1
					0.025		91.2
					0.030		98.4
Com. C	876.02	RhB	8.5	20	0.020	5	64.4
					0.025		84
					0.030		95

## Data Availability

Data can be made available upon request.
